# Anti Mtb Medicinal Plants Database (AMMPDB): A curated database of Indian anti-tubercular medicinal plants

**DOI:** 10.1016/j.jaim.2023.100712

**Published:** 2023-04-28

**Authors:** Jithisree Kanneganti, Usha Mina, Ankita Singh, Anuradha Gautam, Pallavi Somvanshi

**Affiliations:** a School of Environmental Sciences, Jawaharlal Nehru University, New Delhi, India; b Advanced Instrumentation Research Facility, Jawaharlal Nehru University, New Delhi, India; c School of Computational and Integrative Sciences, Jawaharlal Nehru University, New Delhi, India

**Keywords:** Native Indian medicinal plants, Anti-tubercular plants, Phytochemicals, Anti-Mtb database, Computational drug designing

## Abstract

The utilization of medicinal plants for their therapeutic properties has long been a key component of Indian culture. Unique medicinal characteristics can be found in the phytochemicals that are extracted from these plants. Globally, tuberculosis (TB) burden and management are challenged due to the emergence of new resistant strains of *Mycobacterium tuberculosis* (Mtb). This highlights the importance of new drug molecules from diverse sources as well as their innovative management options. In this context, the present study formulated an Anti Mtb medicinal plant database (AMMPDB Ver. 1.1), a manually curated database of native Indian medicinal plants that reported anti-tubercular (anti-TB) activities and their potential therapeutic phytochemicals. This is the first-ever freely accessible digital repository. The current version of the database provides users, with information regarding 118 native Indian anti-tubercular medicinal plants and their 3374 phytochemicals. The database provides the following information: Taxonomical ID, botanical description, vernacular names, conservation status, geographical distribution maps, IC-50 value, phytochemical details which include - name, Compound ID, Synonyms, location in plant part, 2D, 3D structures (as per the availability), and their medicinal uses reported in the literature. The tools section of the database is equipped with sequentially catalogued and hyperlinked open-access tools utilized for computational drug designing. A case study has been incorporated under the contributors section to validate the tools section and the phytochemicals of the database. AMMPDB Ver 1.1 will be serviceable to research in computational drug designing and discovery with effectiveness and ease.

Database URL: https://www.ammpdb.com/

## Graphical abstract

**Figure:**
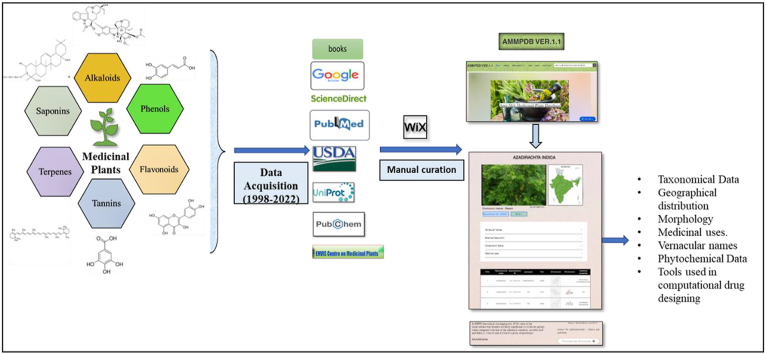

## 1 Introduction

Recording 1.3 million deaths per year across the globe, Tuberculosis (TB) is one of the major public health concerns, affecting primarily the respiratory system [1]. It is a contagious disease primarily attacking the lungs and is caused by a single infectious agent, *Mycobacterium tuberculosis* (Mtb) [2,3]. India tops the list of countries affected by TB with 26.9 lakh cases in 2019 [4]. The government of India has envisioned a TB mukt Bharat (TB-free India) by 2025, five years ahead of the global sustainable development Goal 3 (SDG 3) target 3.3 of 2030. The emergence of new “multi-drug-resistant” (MDR) and “extensively drug-resistant” (XDR) strains of Mtb across the globe, can be attributed to the developed resistance against both first-line and second-line drugs. New drugs and innovative approaches are required for its management to reduce the disease burden.

For centuries, medicinal plants in their pure or crude form have been a source to cure many diseases and continued to be a hope for several emerging strains and diseases. Since time immemorial, this blessing of mother nature has always provided humanity with remedies in the form of local medicine, against diseases [5,6]. Ancient Indian scriptures like “Rig-Veda, Atharvaveda, and Charka Samhita” reveal the plentiful benefits of plants [7]. As listed by WHO universally, 21,000 plants are most widely used for medical purposes. Within India, 2500 species have been identified as traditional medicine for the well-being of mankind [8]. They are of high significance owing to the presence of numerous phytochemicals with high therapeutic value, and their extracts are used for treating various diseases [9]. The enormous diversity of phytochemicals in the plants proved efficient as drugs against several diseases and infections [10]. Many biopharmaceutical companies utilize these medicinal plants on a large scale as conventional medicine [11]. These products have great potential to act as novel drug targets [12,13]. A vast quantity of traditional and phytochemical information about medicinal plants is found dispersed in texts that must be diligently documented and digitalized [14,15]. Databases are principal tools to extract these contents from several printed documents and web systems. Interestingly, within the past few years, enormous efforts have been made toward the inception of databases for medicinal plants [16].

**Fig. 1:**
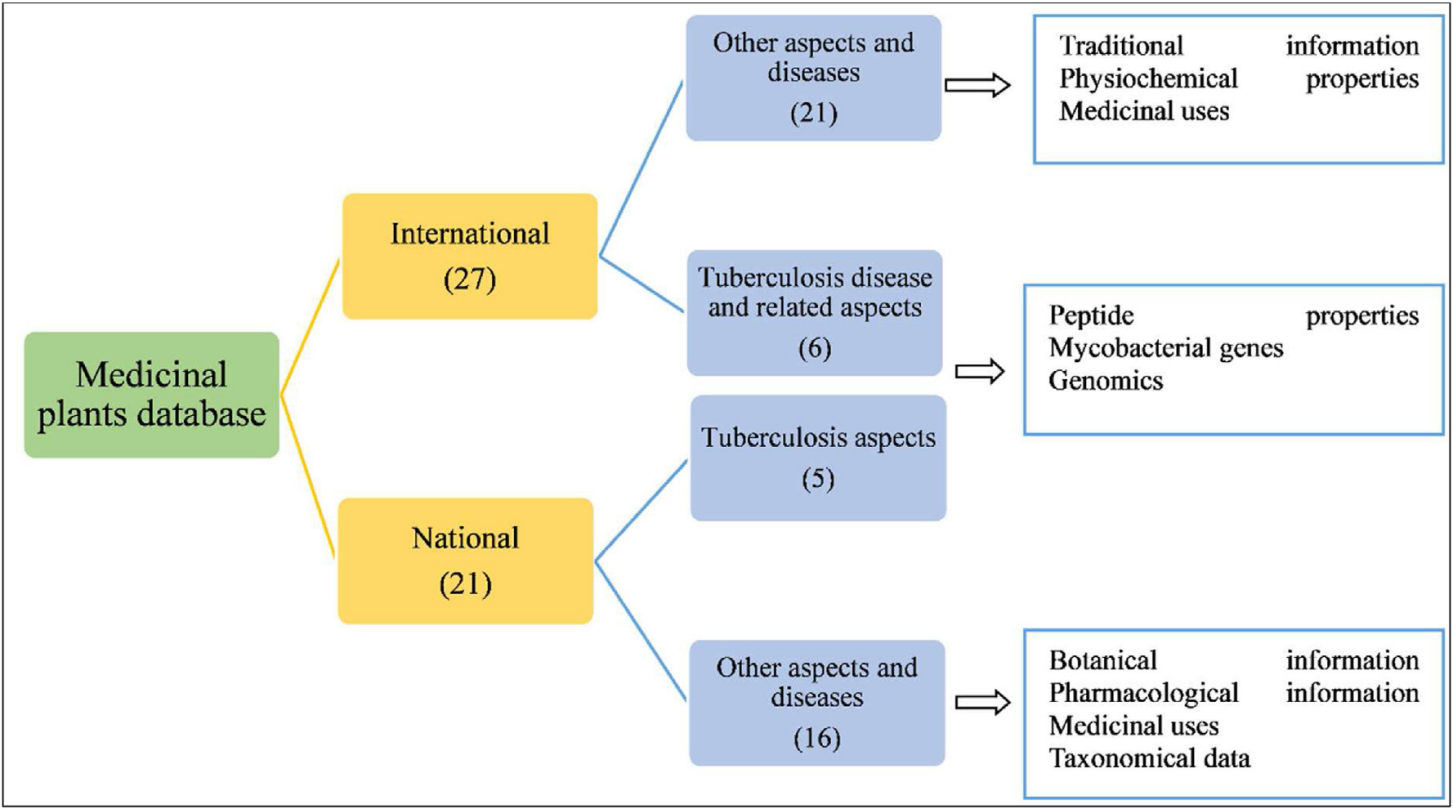
Medicinal plants databases and the attributes reviewed.

**Fig. 2:**
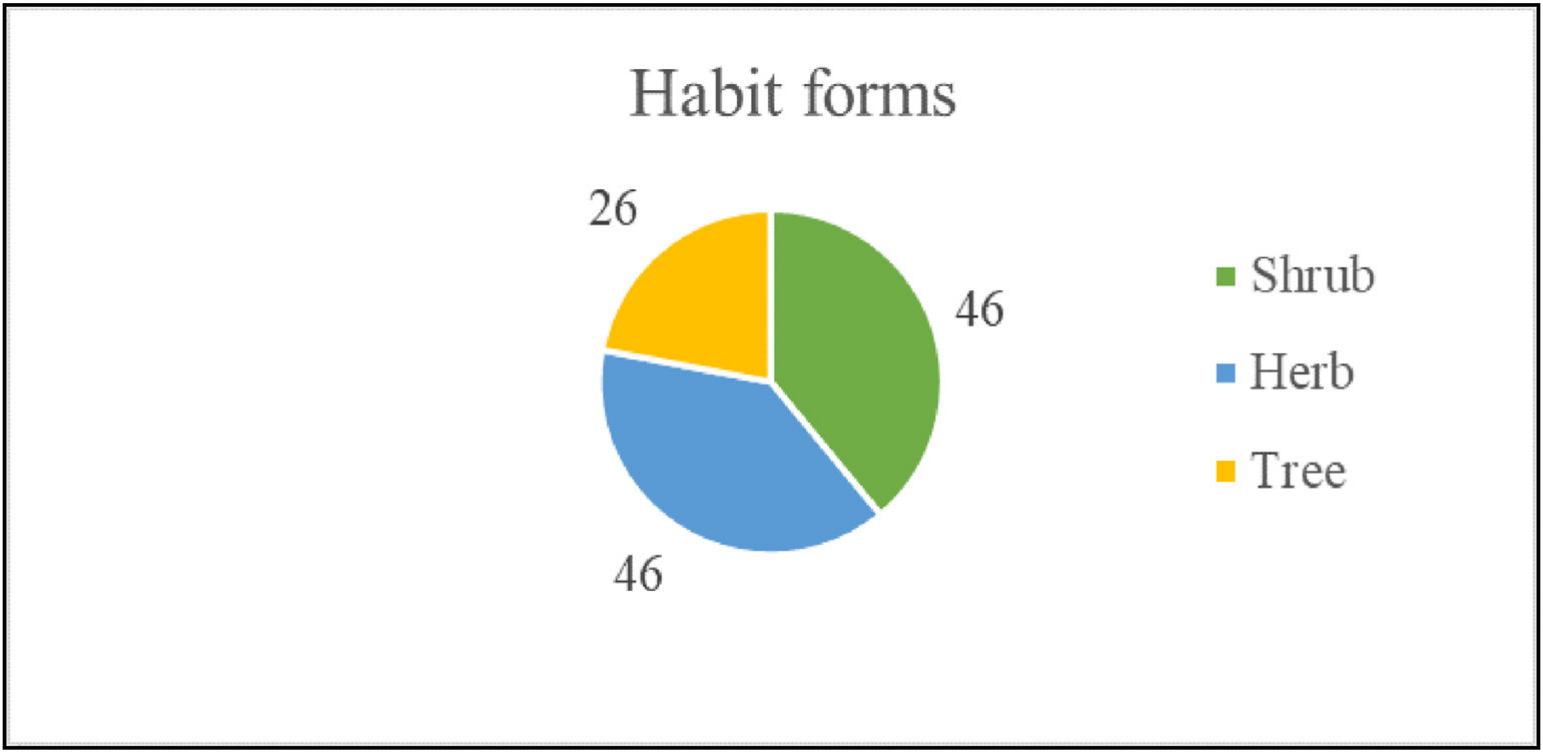
Habit/life forms of anti-tubercular medicinal plants.

**Fig. 3:**
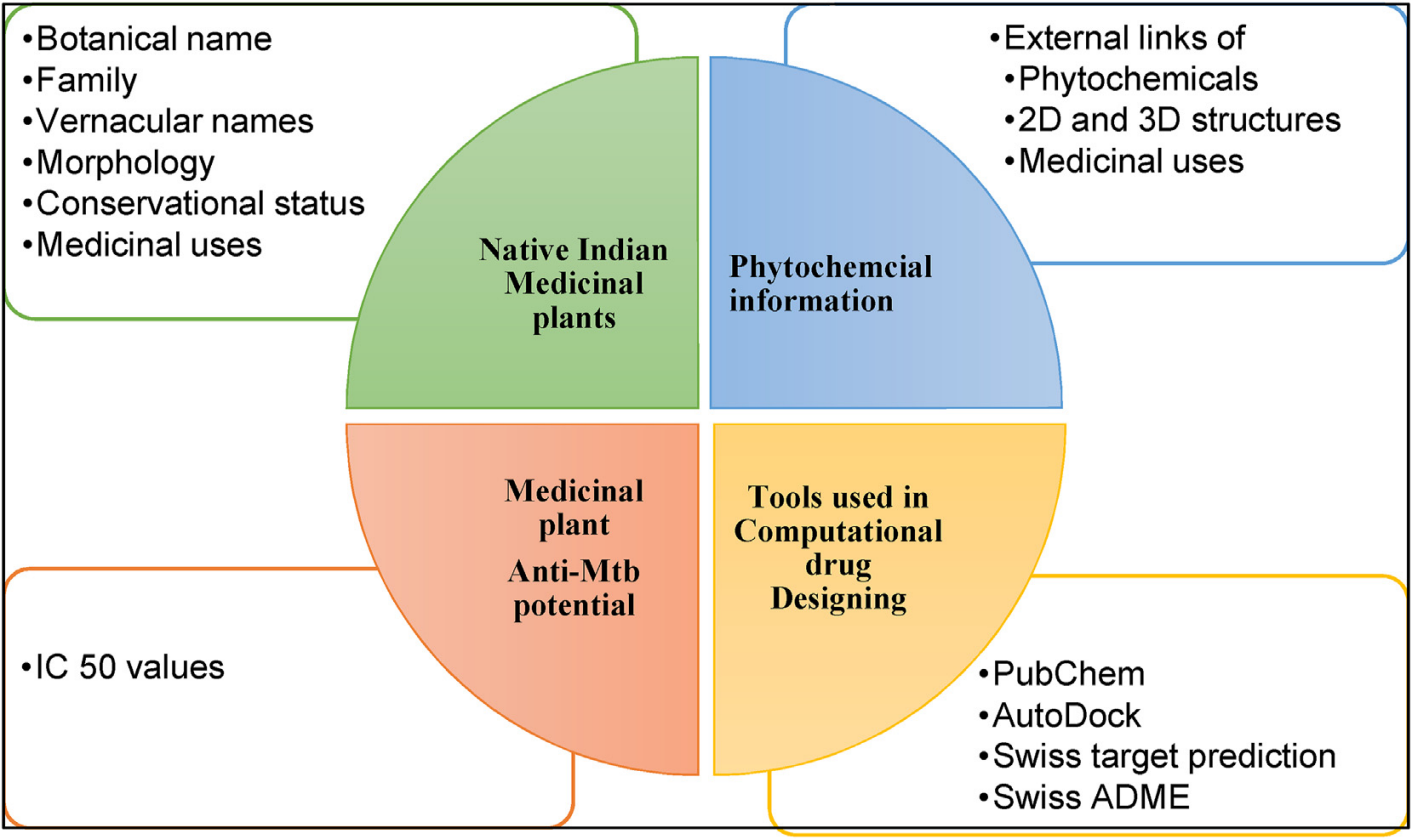
AMMPDB Ver 1.1 layout.

The present study conducted a systematic review of all the available 27 international and 21 national medicinal plant databases published between 1998 and 2022 (Supplementary Table S1, S2, and Fig. 1). Out of which, 11 databases (6 international and 5 national) are addressing tuberculosis disease directly or indirectly (Supplementary Table S3). On reviewing the existing databases, the following limitations were observed – Open access denied, require registration, not user-friendly, only a few databases are based on phytochemical data, limited applications, restricted downloading formats, no database was specific to anti-tubercular Indian medicinal plants reported already in the literature. Some databases are general or region-specific information related to morphological, physiochemical, and phytochemicals of medicinal plants diseases and their specific protein target. For example, the database ‘Phytochemica’ has information related to only 5 plants and 963 phytochemicals. ‘Biophytomol’ provided information on 633 phytochemicals with their inhibition plant extracts for different species of anti-mycobacterium. Other attempts to construct online anti-tubercular databases have largely been limited to cataloging structural information of anti-TB peptides, Mtb genome, and anti-TB drugs. On comparing these databases from the viewpoint of anti-tubercular properties, there have been comparatively little efforts made to construct online databases on native Indian anti-tubercular medicinal plants and their phytochemicals. Hence, the current study primarily focuses on Indian medicinal plants reported as having anti-tubercular properties. Therefore, the first-ever freely accessible digital repository solely created to support discovering novel drug molecules against Mtb for the treatment and management of tuberculosis. AMMPDB Ver 1.1 is a manually curated database of Indian medicinal plants having anti-tubercular (anti-TB) activities with their potential therapeutic phytochemicals. AMMPDB Ver 1.1 was developed to address the gaps in the existing most cited tuberculosis-related medicinal plant databases (Table 1). The current version of the database provides information for 118 anti-tubercular medicinal plants belonging to 62 families representing shrub, tree, and herb habit/life forms (Fig. 2). The database includes 3374 phytochemicals and their details to aid in further computational drug discovery research. The database provides medicinal plant's taxonomical ID, vernacular names, Botanical Descriptions, Conservation status, geographical distribution maps, IC-50 value, phytochemical information (as per availability), and their medicinal uses as reported in the literature (Fig. 3).

**Table 1:** Comparison of AMMPDB with other Anti-mycobacterial databases based on phytochemicals.

S.No	Database	AMMPDB Ver1.1	Phytochemica	Biophytomol
1	Plants	118	5	N.A
2	Plant Families	62	5	188
3	Phytochemicals	3374	963	633
4	Plant-centric list of phytochemicals	Yes	Yes	No
5	Specific to *Mycobacterium tuberculosis*	Yes	No	No
6	Web interface	Yes	Yes	Yes
7	Taxonomical classification of the plant	Yes	No	No
8	Traditional information about the plant	Yes		
8.1	Botanical description	Yes	No	No
8.2	Vernacular names	Yes	No	No
8.3	Conservation status	Yes	No	No
8.4	Medicinal uses	Yes	No	No
9	2D structure of the phytochemicals	Yes	No	Yes
10	3D structure of the phytochemicals	Yes	Yes	Yes
11	Downloadable structure file formats	2D -JPG	2D- N.A	2D –
		3D – SDF,	3D- Jsmol	Mol,SDF
		JPG		3D – Mol, SDF
12	Plants reported under Ayurveda	Yes	No	No
13	IC 50 value of plant	Yes	No	Yes

## 2 Material and methods

### 2.1 Literature review and data acquisition

Data on anti-tubercular plants were manually extracted from various literature sources, such as published and renowned research articles, medicinal plants compendium, and several online databases published during 1998–2022. For the literature survey, different combinations of keywords have been used in mining at Google Scholar, PubMed, Science Direct, and Springer Link, which are available for public access (Table 2).

**Table 2:** List of keywords used in the literature survey.

S. No	Keyword used	Google scholar	Pub Med	Science direct	Springer link
1	Medicinal plants databases	2,45,000	2855	19,135	17,353
2	Indian medicinal plant databases	73,500	119	3487	5321
3	Anti-tubercular Medicinal plants	7920	26	598	230
4	Anti-tubercular databases	6570	57	727	402
5	Anti-tubercular plants and India	5250	46	599	268
6	Anti -tubercular medicinal plants in ayurveda	1350	1	2815	2428
7	Phytochemicals used in the treatment of TB	37,800	58	1028	757

The search criteria resulted in a number of articles. The relevant data from the articles were curated and included in the database after a thorough manual screening of the articles. AMMPDB Ver.1.1 contains taxonomical descriptions depicted using Uniport taxonomy (https://www.uniprot.org/) and tropicos, Geographical distribution maps to provide the user with information about the habitat distribution of the plant across India. They are created using a tool (https://www.mapchart.net/india.html). The vernacular names and botanical descriptions of the plant are provided. Using the IUCN (International Union for Conservation of Nature) red list, each plant's conservational status has been provided. The phytochemical name, compound ID (CID), synonyms, location in plant part, and 2D and 3D structures were retrieved from PubChem and provided in SDF format for the user to download. The plant's half-maximal inhibition capacity (IC50) value is also furnished as per availability.

### 2.2 Database architecture

**Fig. 4:**
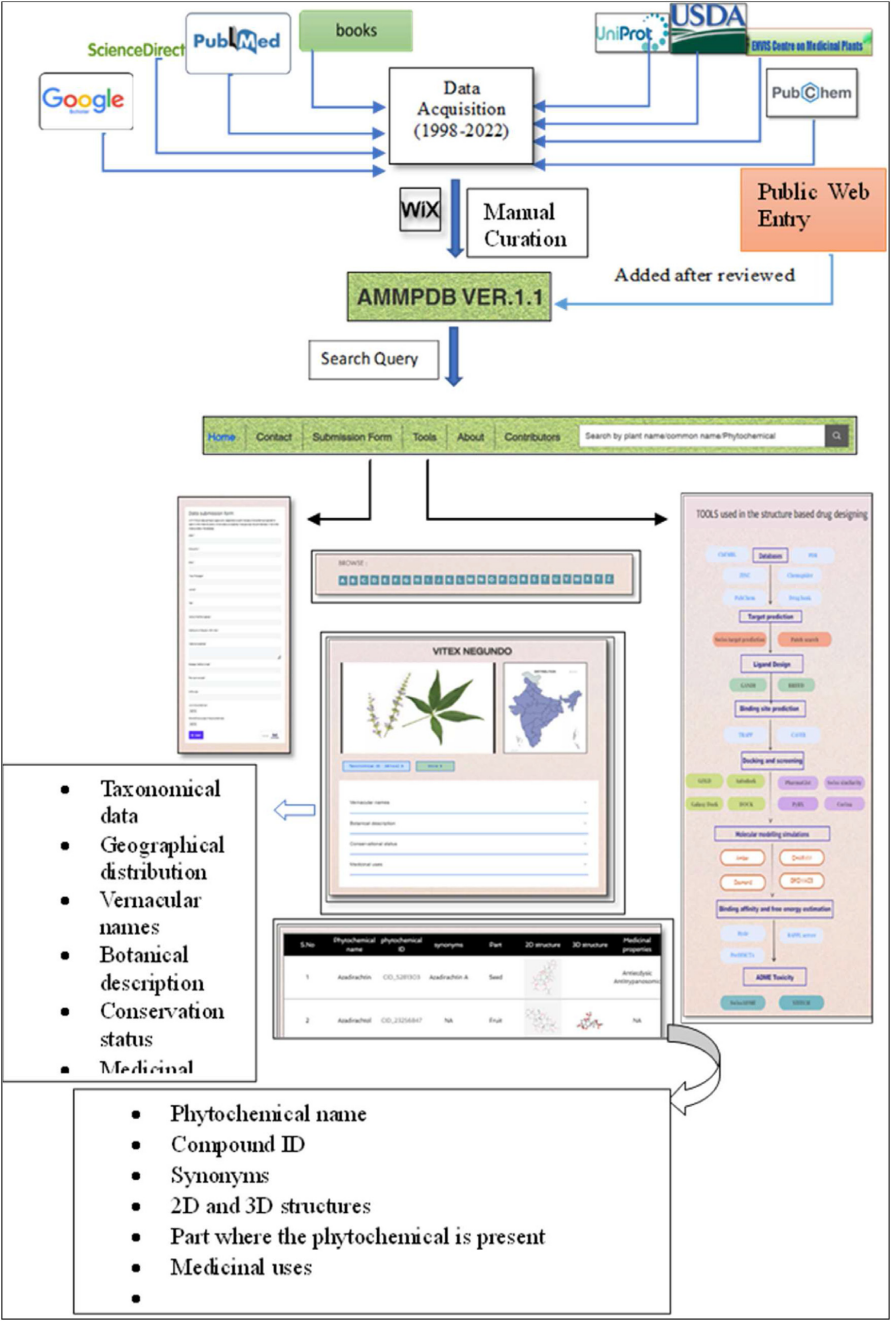
Schematic overview of the AMMPDB ver. 1.1 database construction pipeline.

After thorough mining of the literature, plants of Indian origin reported as having anti-tubercular properties have been selected and reported in the database in a plant-centric approach. The database can be queried through a search box using keywords like plant name, common name, and phytochemical. Every plant has been represented on an individual page with all the relevant aspects along with their phytochemical data. The overview of the database is provided in Fig. 4. The information in the database is separated into distinct segments of the web interface.

#### 2.2.1 Homepage

This page contains a menu bar with some salient features. It contains the following pages: about, contact us, tools, submission form, contributors page, and a search box provided at the top of the page. A concise outline of the database is provided on the homepage for effortless navigation. A graphical representation of the data concerning each medicinal plant is given. Users can also filter plant-related information of their interest by using the scientific name, common name, and phytochemical in the search box provided. A chat box feature is equipped for any further queries.

#### 2.2.2 Plants page

The 118 plants are listed alphabetically. Users can click on the starting alphabet of the plant's botanical name to further access its details.

#### 2.2.3 Plant details

This page includes the following information about the plant: taxonomical ID (Uniprot, tropicos), geographical distribution maps, common name, vernacular names, botanical description, conservation status (IUCN), medicinal properties, and phytochemical data - phytochemical name, their respective compound ID, synonyms, 2D, and 3D structures in downloadable format, and plant parts along with their medicinal properties. The half-maximal inhibitory concentration (IC 50) of the plants already reported in the literature is also provided, along with its source.

#### 2.2.4 Tools page

The freely available open-access tools utilized for computational drug discovery are hyperlinked and arranged sequentially. This tools page acts as a guideline for researchers in the field of computational drug discovery.

#### 2.2.5 Submission form

The user can submit their findings and queries. They will further undergo scrutiny before uploading them to the database. It is open for all the researchers working in the domain for submitting their data to be incorporated through the submission form.

#### 2.2.6 Contributors page

This page gives details of the contributors and their contributions to the database. In the future, all the contributors will be acknowledged.

### 2.3 Database web interface development

AMMPDB Ver 1.1 is user-friendly and openly accessible at www.ammpdb.com. It provides detailed browsing, searching, and export of data. The front end of the database is developed using WIX, a comprehensive cloud-based web designing platform that provides drag-and-drop tools for the customization of the website. The database is well-designed from scratch in an illustrative approach. Once the datasets are filled, they are connected to the table on the front end of the database. The database is mobile-friendly and can be effortlessly accessed from diverse digital devices.

## 3 Result and discussion

**Fig. 5:**
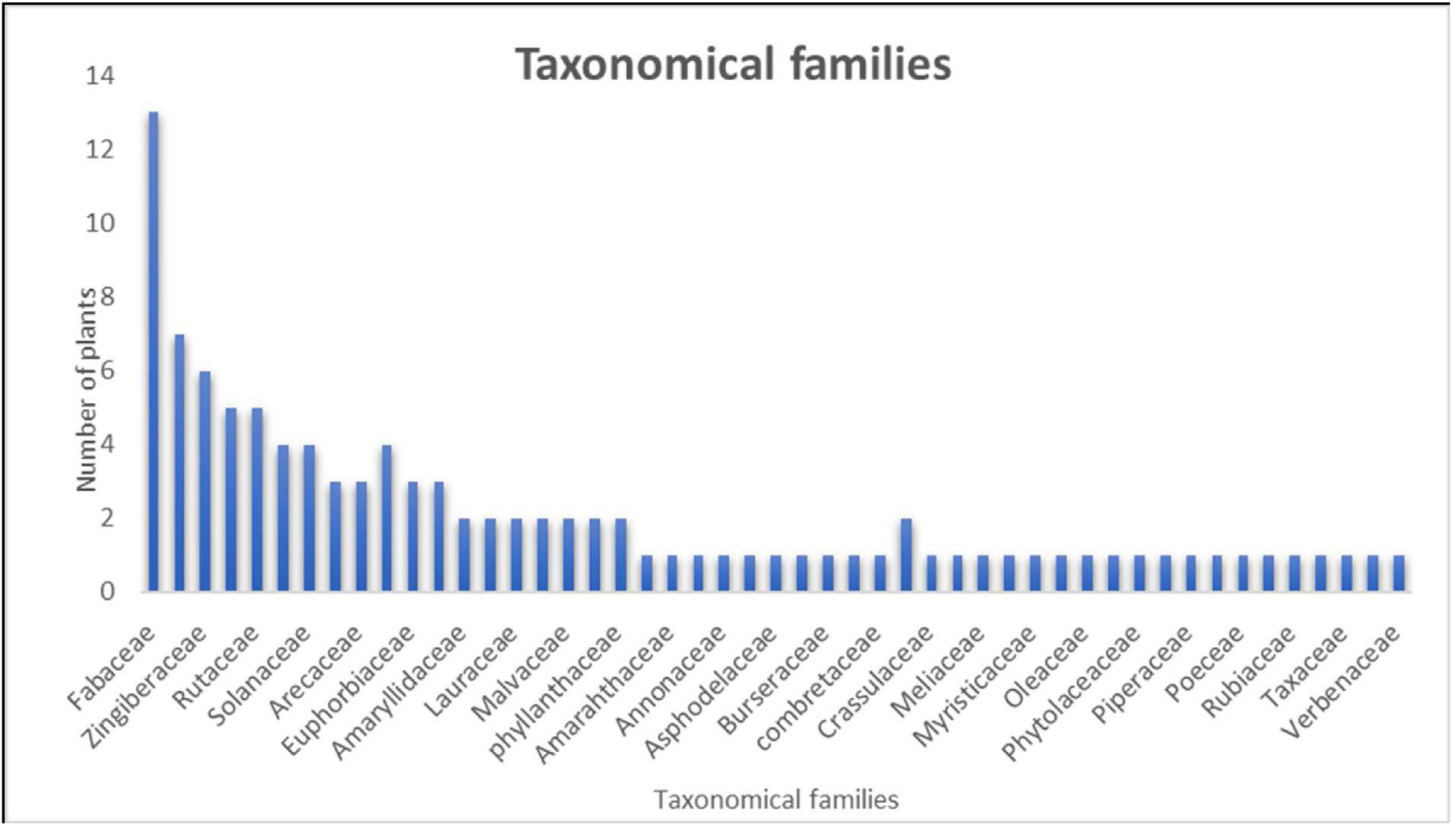
Taxonomical families of Anti-TB medicinal Plants.

**Fig. 6:**
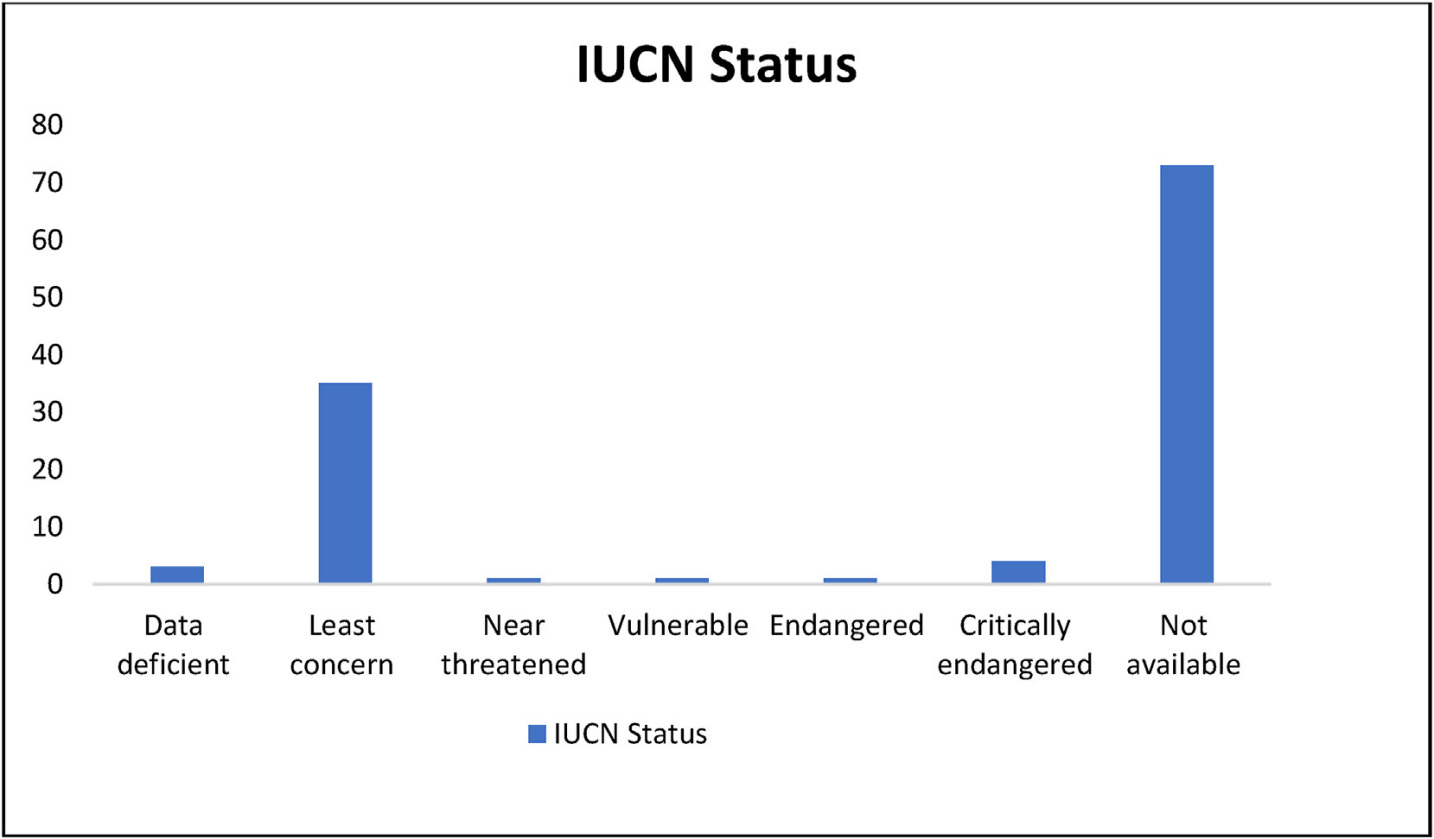
Conservation status of Anti-tubercular Medicinal Plants.

The AMMPDB Ver. 1.1 database contains data of 118 native anti-TB medicinal plants and their 3374 phytochemicals (Supplementary Table S4). It is an open-access resource to identify the potential of phytochemicals from medicinal plants as novel drugs against TB. The AMMPDB Ver. 1.1 database captures information for Indian medicinal plants including taxonomy, vernacular names, geographical distribution, conservation status, medicinal properties, IC-50 value provided for 106 out of 118 plants, and phytochemicals including their 3D and 2D structure in SDF format. The therapeutic benefits of the respective phytochemicals along with the region where they are present can be retrieved. Detailed data like the chemical name and structures can be fetched upon clicking the respective phytochemical. The medicinal plants represented under AMMPDB Ver 1.1 database contain 62 taxonomic families (Fig. 5), among which Fabaceae stands with a maximum (13) number of plants. The IUCN conservation status of each plant is also mentioned as per the availability (Fig. 6).

**Fig. 7:**
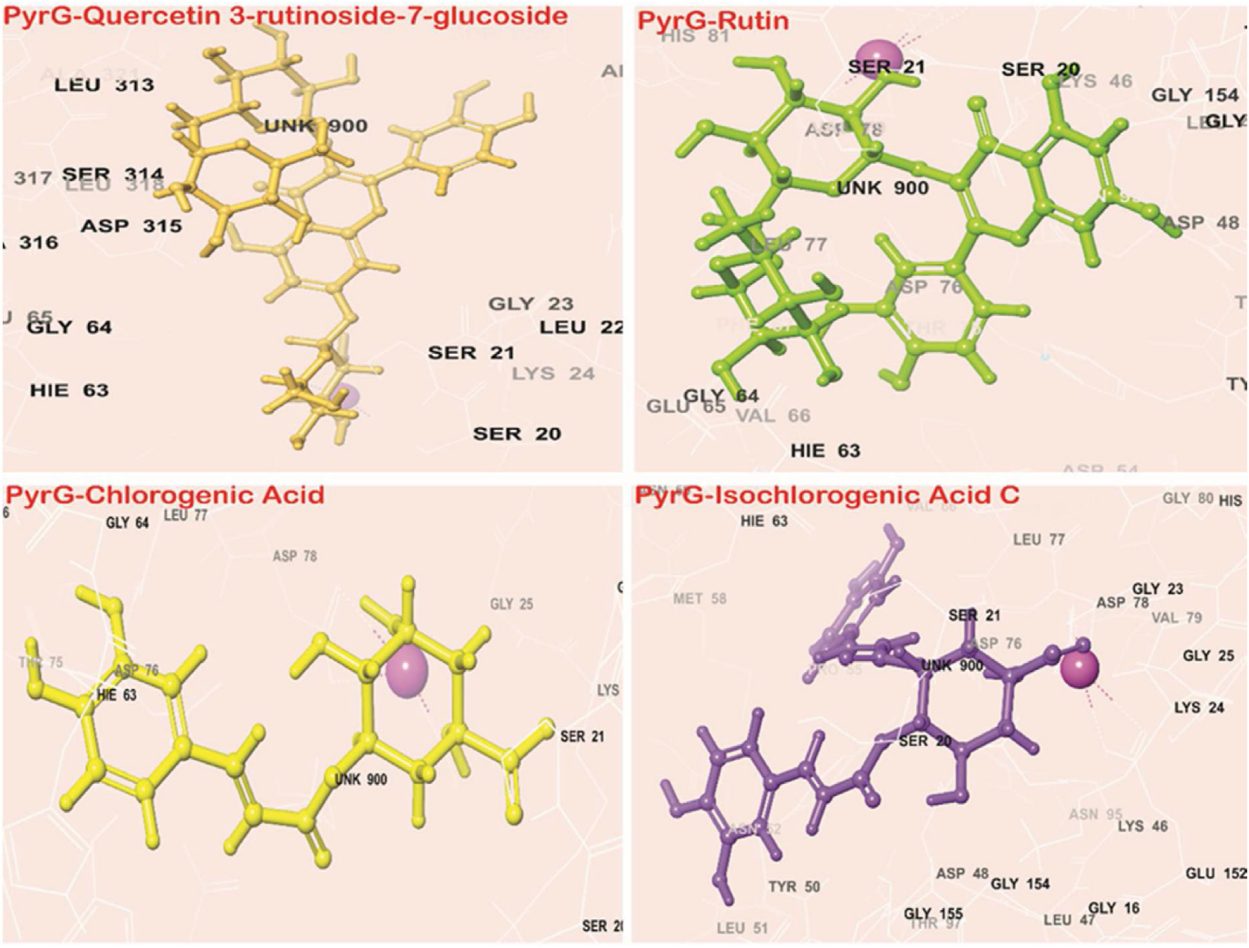
3D interaction diagram of, docked complexes exhibiting interaction with the respective ligands at 4 Å area in the binding pocket of Mtb-PyrG protein.

### 3.1 Case study

To help the beginners (for using the database), an example using a case study is incorporated under the contributors page. AMMPDB (Ver 1.1.) medicinal plants and phytochemicals were used by Singh et al., 2022. The objective of the study is to manage the *Mycobacterium tuberculosis* (Mtb) resistant strains. There is an urgent need to explore novel drug molecules for effective Mtb inhibition. The reactivation of the disease may be minimized using phytochemical-dependent Mtb inhibitors. Which can play a role in developing efficient drugs to counter tuberculosis. In the study, a crucial enzyme PyrG (CTP synthase), is involved in the Mtb biosynthesis pathways to support its growth and is identified as a novel target for developing *Withania somnifera* phytochemical-based drugs to combat resistant Mtb strains.

## 4 Methodology

The study screened medicinal plants listed under the AMMPDB database for novel phytochemicals as potential inhibitors of Mtb PyrG. The steps followed are.

### 4.1 Step 1: Target identification

Target identification (gene/protein) is done through a literature survey. In the present study, identified target CTP synthase PyrG is essential for the biosynthesis of a pyrimidine to support the growth of Mtb and thus possesses a therapeutic target [17,18].

### 4.2 Step 2: Protein and ligand preparation

#### 4.2.1 Step 2.1 3D structure retrieval

Using PDB ID: 4ZDK the crystallographic three-dimensional structure of PyrG target protein was fetched from the RCSB protein Database (https://www.rcsb.org/) in protein data bank (PDB) format having a resolution of 3.4 Å. The Dock Prep tool in UCSF Chimera [19] was used to process and minimized the protein structure under default parameters. All the heteroatoms, native ligands, water molecules, and non-polar hydrogen atoms were removed, followed by adding along with the addition of Gasteiger charges and polar hydrogen atoms from the protein structure, followed by structure minimization.

#### 4.2.2 Step 2.2: 3D structure data of phytochemicals

The 3D structures of 83 phytochemicals of - *W. somnifera* were obtained in SDF format from the AMMPDB database. The ligands were prepared using the AutoDock USCF Chimera tool by adding hydrogen and gasteiger charges under default parameters. The active site residues of PyrG (Ser21, Gly23, Lys24, Gly25, Leu26, Asp78, and Ala253) around the 12 Å region were selected for performing virtual screening on a receptor grid using AutoGrid in USCF Chimera [19].

### 4.3 Step 3: Structure-based virtual screening and redocking

The top compounds with elevated negative docking scores having a high binding affinity with lower binding energy are identified using the Structure-based virtual screening (SBVS) computational technique. Out of the 83 phytochemicals of *W. somnifera*, those possessing notable binding energy were selected as influential inhibitors of PyrG using MtiOpenScreen [20]. Redocking of proteins with the selected compounds was performed by Autodock Vina and Mti-Autodock [20] under default parameters. 2D and 3D interaction figures were generated using the Maestro tool of the Schrödinger suite (academic version).

### 4.4 Step 4: Molecular dynamic simulation

The dynamic stability and intermolecular interactions of the identified compounds inside the active site residues of the Mtb PyrG protein were examined using molecular dynamic (MD) simulations. The MD system for top-docked complexes was prepared, neutralized, minimized, and stimulated using Desmond, an academic version of the Schrödinger Suite and Gromacs.

Post-dynamics simulation analysis.

### 4.5 Step 5: Pharmacokinetic and toxicity

Additionally, pharmacokinetic characteristics such as absorption, distribution, metabolism, and excretion (ADME) and Toxicity(T) were examined for the top compounds (ADME), and Toxicity (T) was analyzed using SwissADME (http://www.swissadme.ch/) [21].

## 5 Result

Molecular docking identifies the top four phytochemicals of *W. somnifera* i.e., Quercetin 3-rutinoside-7-glucoside, Rutin, Chlorogenic acid, and Isochlorogenic acid C with the substantial binding score, as potential Mtb-PyrG inhibitors (Table 3) (Fig. 7). The stability of docked complexes and the drug-likeness of certain compounds are further supported by molecular dynamics simulations and ADME analysis, respectively.

**Table 3:** Docking score of all four selected potential compounds using different Tools.

S.No.	Drug	MTiAutoDock 4.2.6	AutoDock Vina
1.	Quercetin 3-Rutinoside-7-Glucoside	−9.71	−9.1
2.	Rutin	−9.53	−8.4
3.	Chlorogenic-Acid	−7.37	−7.4
4.	Isochlorogenic-Acid-C	−7.30	−8.4

## 6 Conclusion

AMMPDB Ver1.1 is a comprehensive, open-source database developed to compile data on indigenous anti-tubercular Indian medicinal plants and their phytochemicals. The database contains 118 anti-tubercular medicinal plants belonging to 62 families and their 3374 phytochemicals. It provides the user with medicinal plant details along with their phytochemical 3D structures for drug designing to determine their therapeutic potential against Mtb targets. The freely accessible drug profiling tools provided in the database will guide beginner researchers in structure-based drug design. With the escalation seen in the field of science, it becomes crucial that the biological community promotes the use of databases to document and digitize the data, providing easy access and efficient management of the data. To encourage user interaction, the quality of the data and new findings will be continually added to this edition of the database. Researchers working on medicinal herbs, computational drug designing, and discovery will find the usage of AMMPDB Ver 1.1 valuable against the emerging Mtb-resistant strains.

## 7 Limitations

The current version of the database is restricted to only 118 anti-tubercular Indian medicinal plants, with only 3374 phytochemicals available. The information available in this database is provided only in English language at present. In the further upgrade, the database will be accessible in other languages with additional plants.

## Source(s) of funding

This research did not receive any specific grant from funding agencies in public, commercial, or not-for-profit sectors.

## Declaration of competing interest

The authors report no conflict of interest. The authors alone are responsible for the content and writing of the article.
