# Supplementary material for: Anti Mtb Medicinal Plants Database (AMMPDB): A curated database of Indian anti-tubercular medicinal plants
Source: J Ayurveda Integr Med. 2023 Apr 28;14(2):100712. doi: 10.1016/j.jaim.2023.100712 (PMC10172712; doi:10.1016/j.jaim.2023.100712)
Supplement: Multimedia component 1 [file mmc1.docx]

Table S1: Architectural components of international plant databases

| S. No | Database name | Attributes/components provided in the database | References |
| --- | --- | --- | --- |
| 1 | Biofacquim | - Taxonomy, - Physiochemical properties, - Publication. | [22] |
| 2 | Dr.Duke’s Phytochemical & Ethnobotanical Database | - Phytochemicals, - Medicinal uses, - and references. | - |
| 3 | ETM-DB | - Phytochemicals, - target genes, - proteins. | [23] |
| 4 | Herbal DB 2.0 | - Chemical compounds | [24] |
| 5 | Herbmedpro | - Scientific name - Taxonomy - Traditional, - folk use formulas and blends. | - |
| 6 | KNApSack | - Metabolite info - Molecular weight, - Molecular formula - C_ID - CAS ID | [25] |
| 7 | Malaysian herbal monograph | - Plant morphology - Thin layer chromatography - High - performance liquid chromatography - Chemical constituents - Toxicology studies - Medicinal properties | - |
| 8 | Mapsdatabase | - Phytochemical constituents - structures | [26] |
| 9 | Medicinal herbs and plants monographs | - Taxonomic - Pharmacology - Cultivation |  |
| 10 | MedPhyt | - Pharmaceutical information - toxicological | [27] |
| 11 | Metacrops | - Metabolic pathways - Crop metabolism | [28] |
| 12 | MPB – 3.1 (Medicinal plant of Bangladesh) | - Scientific name - Taxonomic - Medicinal uses | [29] |
| 13 | MPDB 2.0 | - Taxonomy - phytochemical data - vernacular names - compounds - Parts used | [30] |
| 14 | PfaF- Plants for a future | - General info - Characteristics - Cultivation - Medicinal use | - |
| 15 | Plants database | - Taxonomic info - Distribution data - Characteristics - Uses |  |
| 16 | PMN (plant metabolic network) 15 | - Overview - Taxonomic | [31] |
| 17 | The Plant list | - Taxonomical information - Synonyms | [32] |
| 18 | Traditional Chinese Medicine Integrated Database (TCMID) | - Pharmacology - Medicinal uses | [33] |
| 19 | Tropics | - Taxonomical information - Morphology - Chromosome counts - Authors and publication details |  |
| 20 | Vietherb | - Morphology - Taxonomy - Metabolites - Therapeutic effects. - Geographical locations | [34] |
| 21 | World flora online | - Taxonomic and morphology |  |

Table S2: Architectural components of national plant databases

| S.No | Database name | Architectural components in the database | References |
| --- | --- | --- | --- |
| 1 | CCRAS-Central council for research in ayurvedic science | Botanical names  vernacular names  uses |  |
| 2 | Database for ethnomedicinal plants of Western Ghats | Vernacular names  Description  Pharmacology  Chemical composition  Uses | [35] |
| 3 | DEPTH  (Database on endemic plants at Tirumala hills) | Vernacular name  Taxonomy  Botanical description | [36] |
| 4 | Dravyaguna database | Botanical information  Ayurvedic information |  |
| 5 | FRLHT ‘S ENVIS | Botanical description of plants  Herbarium |  |
| 6 | IMPLAD  (Indian Medicinal Plants Database) | Botanical information  Distribution  GIS  Traditional knowledge. | [37] |
| 7 | IMPPAT  Indian medicinal plants, phytochemistry and therapeutics | Common name  Taxonomy  Phytochemical: name  Compound ID  2D/3D Structure  SMILES  InChlkey  Physiochem prop  Drugability prop  ADMET | [38] |
| 8 | Indiabiodiversity.org | Overview of the plant   - - Description   - Distribution   - Uses and management   - References   - Taxonomy | - |
| 9 | InDiamed | - chemical, - pharmacological, - biochemical, - geographical information of the medicinal plant | [39] |
| 10 | Indian medicinal plants database | - Taxonomical and bibliography |  |
| 11 | Med-pdb | - Botanical name - Taxonomical - Morphological - Geographical location - Administration - Active compounds - Biological targets PMID | [40] |
| 12 | MPD.aripune | - Plant information, - Location-specific data, - species-specific data, - distribution maps, trade resources, and Ethno resources. species index, plant information, location specific data, species -specific data, distribution maps, trade resources, and Ethno - resources |  |
| 13 | Nemedplant | Parts used constituents and description, structures references: title, author, journals PubChem | [41] |
| 14 | Phytochemica | Physiochemical properties ADMET properties Plant-derived molecules Phytochemicals Statistical distribution | [42] |
| 15 | Plant molecular taste DB | A Database of Taste Active Phytochemicals | [43] |
| 16 | Umpdb  (Uttarakhand medicinal plants database) | Traditional info and uses Genomic information External links and platforms Chemical info and literature | [44] |

Table S3: List of a few antitubercular databases reviewed

| S.No | Database name | Description | Hyperlink | References |
| --- | --- | --- | --- | --- |
| 1 | AntiTbPdb  (Anti tubercular peptides) | Structural information and properties of peptides like physiochemical properties. | <https://webs.iiitd.edu.in/raghava/antitbpdb/index.html> | [45] |
| 2 | TB Drug target Database | Antitubercular drugs, and target proteins for the treatment of TB. | <https://www.bioinformatics.org/tbdtdb/userguide.htm#Heading1> | **-** |
| 3 | BioPhytMol | Anti-mycobacterial Phyto molecules and plant extracts | <https://ab-openlab.csir.res.in/biophytmol/> | [46] |
| 4 | Biohealthbase | Public bioinformatics database | <https://www.ncbi.nlm.nih.gov/pmc/articles/PMC2238987/> | [47] |
| 5 | CDD TB | Web-Based database solution | <https://www.collaborativedrug.com/cdd-receives-new-tb-funding/> | **_** |
| 6 | GenoMycDB | Comparative analysis of mycobacterial genes | <https://www.ncbi.nlm.nih.gov/pubmed/16755503> | [48] |
| 7 | TBrowse | General purpose browsing mechanism for table-oriented data | <https://www.itlnet.net/programming/program/Reference/c53g01c/ng15a315.html> | _ |
| 8 | TDR | Rapid identification and prioritization of molecular targets for drug | <https://tdrtargets.org/> | - |
| 9 | TBDB | Comparative genomics and microarray analysis | <https://www.hsls.pitt.edu/obrc/index.php?page=URL1234384552> | [49] |
| 10 | Web TB | Search and browse of TB genomes and structures | <https://webtb.org.cutestat.com/> | - |
| 11 | TuberQ | A Mycobacterium tuberculosis protein druggability database | <http://tuberq.proteinq.com.ar/> | [50] |

Table S4: List of selective Indian Anti-tubercular Medicinal plants of AMMPDB Ver.1.1

| S. No. | Plant Species and  Family | Ayurveda Name | Part used | Other properties | Reference |
| --- | --- | --- | --- | --- | --- |
| 1 | *Abrus precatorius* L.  (Leguminosae) | Gunja | Seeds | Anti-malarial and anti-convulsant | [51] |
| 2 | *Acacia senegal* (L.) Willd.  (Leguminosae) | Kher or khor | Leaves | Leaves are used to treat cough, respiratory tract infections, bronchitis | [51] |
| 3 | *Acalypha indica*  Euphorbiaceae | Kuppi | Leaves | Antibacterial, used  in bronchitis,  asthma | [3] |
| 4 | *Acorus calamus* L.  (Acoraceae) | Vacha, Ugargandha | Rhizome | Neuroprotective, Anti-arthritic,  Anti-inflammatory, Antibacterial,  Anti-hypertensive,  Sedative, Analgesic | [51] |
| 5 | *Adhatoda vasica* Nees  (Acanthaceae) | Vaasaa | Leaves, flowers and roots | Expectorant (used  in bronchial asthma) | [3] |
| 6 | *Aegle marmelos* L.  (Rutaceae) | Bilwa or bael | Plant | Antiviral  Anticancer  Antipyretic | [52] |
| 7 | *Allium cepa* L.  (Amaryllidaceae) | Palaandu | Bulbs | Antibiotic,  antibacterial,  antisclerotic,  anticoagulant | [3] |
| 8 | *Allium sativum* L.  (Amaryllidaceae) | Lashuna | Bulbs | Antibiotic, fungicide,  bacteriostatic,  anthelmintic,  antithrombic,  hypotensive,  hypoglycaemic,  hypocholesterolaemic | [3] |
| 9 | *Aloe vera (L.) Burm.f.*  (Xanthorrhoeaceae) | Ghritkumaar-  ika | Bulbs | Antioxidant, antibacterial, antiviral, antiseptic | [3] |
| 10 | *Alpinia galanga (L.) Willd.* (Zingiberaceae) | Kulanjan | Rhizome | Immune-modulator, anti-microbial, anti-diabetic, anti-oxidant | [53] |
| 11 | *Alstonia scholaris (L.) R. Br.* (Apocynaceae) | Saptaparna | Bark | Antipyretic, digestive | [76] |
| 12 | *Amaranthus tricolor (L.)*  (Amaranthaceae) | Lal sag | Whole plant | Used to treat cough | [52] |
| 13 | *Andrographis paniculata* Nees  (Acanthaceae) | Kalmegh | Plant extract | Used to treat sore throat and leprosy | [54] |
| 14 | *Annona muricata*  ( Annonaceae) | Lakshmanaphala | Bark  Leaves  Fruits  Flowers  Roots | Antibacterial  Antiviral | [54] |
| 15 | *Aquilaria agallicha*  *(* Thymelaeceae) | Agaru | Plant | Anti-inflammation | [55] |
| 16 | *Asparagus racemosus* Willd. (Asparagaceae) | Satavari | Roots | antitussive, antineoplastic, galactologue, antidiarroheal, and as immunomodulant | [51] |
| 17 | *Avicennia alba*  (Acanthacea) | Kala bani | Fruit  Seed | Antibacterial | [56] |
| 18 | Azadirachta indica A.Juss.  (Meliaceae) | Neem | Leaves, bark and fruit | antibacterial, antiviral, sedative antifungal, anti-diabetic | [51] |
| 19 | *Barleria lupilina* LINDL  (Acanthaceae) | Kanta | Leaves  and  Stem | Antibacterial  and  antioxidant | [52] |
| 20 | *Bauhinia variegata* L.  (Leguminosae) | Kachnar | Stem, flowers, roots | Anti-microbial, Anti-oxidant, Antidiabetic, Anti-carcinogenic, Insecticidal | [51] |
| 21 | *Berberis aristata* DC. (Berberidaceae) | Daruharidra | Roots | antibacterial, antiperiodic, antidiarrheal and anticancer | [53] |
| 22 | *Borassus flabellifer* L. (Arecaceae) | Tala | Roots | Analgesic, antipyretic effects, anti-inflammatory, anti-helminthic and diuretic | [53] |
| 23 | *Caesalpinia pulcherrima* (L.) Sw. (Leguminosae) | Padangam | Leaves and flowers | Laxative,  antipyretic | [3] |
| 24 | *Calotropis gigantea*  ( Apocynaceae ) | Rakta arka and shveta arka | Plant | Antibacterial | [57] |
| 25 | *Camelia sinensis*  ( Theaceae) | Syamaparni | Leaf | Antioxidant | [58] |
| 26 | *Cannabis sativa.L*  ( Cannabaceae) | Bhanga | Flower buds, leaves and stems | Used in treatment of asthama,insomnia | [59] |
| 27 | *Canscora decussata* (Roxb.) Schult. & Schult.f.  Gentianaceae | Daakuni | Roots | Anticonvulsant,  CNS depressant, anti-inflammatory, hepatoprotective | [3,60] |
| 28 | *Capsicum annum .*L  (Solanaceae) | Shimla mirch | Fruit | Antioxidant | [52] |
| 29 | *Cedrus deodara*(Roxb. ex D.Don) G.Don  Pinaceae | Devadar | Leaves | Anti-inflammatory, Antiseptic  Antispasmodic  Antiviral Astringent  Carminative, Diaphoreti | [51] |
| 30 | *Ceiba pentandra*  (Bombacaceae) | Semal | Stem  Bark | Antibiotic | [61] |
| 31 | *Centella asiatica* (L.) Urb.  Apiaceae | Mandukaparni | whole plant | anti-inflammatory, antiproliferative, anticancer, antioxidant, antiulcer, wound healing, | [62] |
| 32 | *Cinnamomum tamala* (Buch.-Ham.) T.Nees & Eberm.  (Lauraceae) | Tejpatra, tamapatra | Bark | Carminative  Anti-spasmodic Immunostimulant Antioxidant  Anti-Diabetic  Anti-Inflammatory Anticancer Antimicrobial | [53] |
| 33 | *Cinnamomum zeylanicum* Blume (Lauraceae) | Tvak, Darusita Daruchini | Bark | anti-microbial,  anti-parasitic,  anti-oxidant, carminative, antiseptic and astringent | [53] |
| 34 | *Citrullus colocynthis* (L.) Schrad.  (Cucurbitaceae) | Indravaruni | Fruit, root | antibacterial, purgative, antifungal, and antioxidant | [54] |
| 35 | *Citrus aurantium amara .L*  (Rutaceae) | Lime | Fruit | Antibacterial,  anticancer,  antidiabetic, antifungal and antioxidant | [52] |
| 36 | *Clitoria ternatea .*L  (Leguminosae) | Aparajita | Whole plant | Anxiolytic and antidepressant | [52] |
| 37 | *Colebrookea oppositifolia* Sm. (Lamiaceae) | Binda | Leaves, fruits and roots | Anti-inflammatory | [3,60] |
| 38 | *Colocassia esculenta*  (Colocasieae) | Arbi | Leaf | Antifungal and anticancer | [52] |
| 39 | *Commiphora mukul* (Hook. ex Stocks) Engl.  (Burseraceae) | Guggul | Resin | antimicrobial, anti-inflammatory, anti-carcinogenic, carminative, antiseptic | [64] |
| 40 | *Costus igneus*  ( Costaceae) | Bandugapushpa | Stem and root | Lower blood glucose levels | [65] |
| 41 | *Cressa cretica*  (Convolvulaceae) | Rudravanti | Plant | Anthelmintic and antidiabetic | [57] |
| 42 | *Croton macrostachyus*  ( Crotoneae ) | Woodland croton | Bark, twigs, fruits,  Leaves,Roots and seeds | Antimicrobial | [66] |
| 43 | *Cryptolepis sanguinolenta*  (Apocynaceae) | Tamalaki | Root | Anti-inflammatory,  antibacterial,  anti-fungal and  anti-malaria | [51] |
| 44 | *Cuminum cymium*  (Umbellifers) | Jeeraka | Seeds | Anticancer and antidiabetic |  |
| 45 | *Curcuma longa* L.  (Zingiberaceae) | Haridra | Rhizome | antioxidant,  anti-inflammatory, anti-mutagenic, antimicrobial, and anticancer | [67] |
| 46 | *Cymbopogon citratus*  ( Poaceae) | Bhutrin | Leaves and whole plant | Antispasmodic  Antispetic | [68] |
| 47 | Dactylorhiza hatagirea  (Orchidaceae) | Salampunja | Tubers and leaves | Used in treatment of diarrhoea, stomachache | [69] |
| 48 | *Datura metel*  (Solanaceae) | Dhatura | Leaves,  seeds | Antitumor,  anti-rheumatic and anti-inflammatory and its flowers | [70] |
| 49 | *Derris indica .*L  (Leguminosae) | Karanj | Stem and root | Antifungal | [52] |
| 50 | Desmodium gangeticum  (Fabaceae) | Shalparni | Roots and whole plant | Anti-inflammatory  Analgesic  Diuretic | [71] |
| 51 | Dioscorea bulbifera  (Discoreaceae  ) | Varanikhand/Varani | Bulbils | Diabetes  Cancer | [72] |
| 52 | *Eclipta prostrata* (L.) L.  (Compositae) | Bhringaraja | Roots | Antimicrobial, Antioxidant, Carminative, Detoxifying, Neuroprotective | [53] |
| 53 | *Elettaria cardamomum* (L.) Maton  (Zingiberaceae) | Sukshamaila | Seed pods | antimicrobial, antioxidant, anti-inflammatory | [53] |
| 54 | *Emblica officinalis* Gaertn.  (Phyllanthaceae) | Amalika | Fruits | antioxidant, immune modulatory, antipyretic, analgesic, anti-inflammatory | [53] |
| 55 | *Ficus carica.*L  (Moraceae) | Anjir | Leaves | Antispasmodic and anti-inflammatory | [52] |
| 56 | *Flacourtia jangomas* (Salicaceae) | Vikankata | Leaves, roots, bark and fruits | Anticholerin | [51] |
| 57 | *Foeniculum vulgare Mill.*  (Apiaceae) | Misreya | Plant | Anti-inflammatory,  Analgesic and diuretic | [73] |
| 58 | *Garcinia polyantha*  ( Clusiacea) | Vrikshamla | Plant | Antidiabetic and antioxidant |  |
| 59 | Gloriosa superba  (Colchicaceae) | Langali | Sap, tubers and seeds | Treat acne,  Anthelmintic | [74] |
| 60 | *Glycosmis pentaphylla*  (Rutaceae) | Ban nimbu | Flowers and fruit | Anticancer ,antibacterial and antifungal | [52] |
| 61 | *Glycyrrhiza glabra* L.  Leguminosae | Mulethi | Roots | Antimicrobial, antioxidant,  anti-inflammatory, antitussive, antidiabetic, antiviral, anticancer, antimutagenic | [75] |
| 62 | *Hedychium ellipticum*  (Zingiberaceae) | Kapur or kachri | Rhizome | Anti-inflammatory | [52] |
| 63 | *Hemidesmus indicus* (L.) R. Br. ex Schult.  Apocynaceae | Sveta Sariva, Anantmool | Roots | demulcent, alterative,  astringent, diaphoretic, diuretic, tonic, anti-pyretic, and blood purifier | [53] |
| 64 | *Hibiscus rosa-sinensis .*L  *(Malvaceae)* | Gudhal | Leaves | Antispasmodic ,antibacterial and antifungal | [52] |
| 65 | *Holarrhena antidysenterica* (Roth) Wall. ex A.DC.  Apocynaceae | Kurchi, Kutaj, Kutaja & Vatsak | seeds | Anti-dysenteric, antidiarrheal, and anti-amoebic | [76] |
| 66 | *Hyptis suaveolens .*L  (Lamiaceae) | Vilayti | Whole plant | Anti-inflammtory,  antispasmodic and diuretic | [52] |
| 67 | Inula racemosa  (Asteraceae) | Puskara | Rhizome s and roots | Anti-inflammatory  Diuretic | [78] |
| 68 | Ipomea turpethum  (Convolvulaceae) | Trivrit | Barks  Leaves  Roots | Anti-inflammatory | [52] |
| 69 | *Jasminum sambac .*L  (Oleaceae) | Chameli | Leaves | Anti-inflammatory,  antiseptic and analgesic | [52] |
| 70 | *Jatropha curcas* L.  Euphorbiaceae | Darvanti | Stem Bark | antimicrobial, anti-cancer and anti-HIV activity | [79] |
| 71 | *Kaempferia galanga.*L  (Zingiberaceae) | Chandramoolika | Plant | Antipyretic and diuretic | [51] |
| 72 | *Kalanchoe integra* (Medik.) Kuntze (Crassulaceae) | Parnabija | Leaves | Hypotensive,  Antiarrhythmic. | [3] |
| 73 | *Lantana camara .*L  (Verbanaceae) | Raimuniya | Plant | Anti-spasmodic,  anti-tumor,  carminative and  anti-inflammatory | [52] |
| 74 | *Lawsonia inermis* L.  Lythraceae | Madayanti | Leaves, roots, stem bark, flowers and seeds | antibacterial, antifungal, antiparasitic, antiviral, anticancer, antidiabetic, anti-inflammatory, antifertility | [80] |
| 75 | *Leptadenia reticulata* (Retz.) Wight & Arn.  Apocynaceae | Jivanti, Hemavati | leaves | Anti-microbial, revitalizing, rejuvenating, and lactogenic | [51] |
| 76 | *Mallotus philippensis* (Lam.) Müll.Arg.  Euphorbiaceae | Kampillaka | Glandular trichomes and hairs of fruit | Purgative,  anthelmintic, styptic | [3] |
| 77 | *Mimosa rubicaulis .*Lam  (Fabaceae) | Shikanta | Leaf | Anti-bacterial and antioxidant | [52] |
| 78 | *Mimosa pudica* L.  Leguminosae | Laajavanti | Leaves and roots | Astringent,  alterative | [3] |
| 79 | M*orinda citrifolia* L.  Rubiaceae | Ashyuka | Leaves, roots and fruits | Antileucorrhoeic, antidysenteric  emmenagogue | [3] |
| 80 | *Morus alba .*L  (Moraceae) | Shahtoot | Fruit and leaves | Antioxidant and antiseptic | [52] |
| 81 | *Murraya paniculate .*L  (Rutaceae) | Kamini | Leaves | Antidote and astringent | [52] |
| 82 | *Myristica fragrans* Houtt.  Myristicaceae | Jatiphala | Seed | anticancer, antidepressant, antidiabetic, antiobesity, antiinflammatory, analgesic, antimicrobial, antioxidant | [3] |
| 83 | *Myrtus communi*s L.  Myrtaceae | Muuraddaan | Fruits | Antimicrobial,  antiparasitic  antiseptic | [3] |
| 84 | Nigella sativa  (Ranunculaceae) | Kalonji | Seeds  Leaves | Antihypertensive  Antibacterial | [81] |
| 85 | *Ocimum sanctum* L.  Lamiaceae | Tulasi | Leaves, flowers and seeds | Carminative,  stomachic,  antispasmodic,  antiasthmatic,  antirheumatic,  expectorant,  hepatoprotective,  antiperiodic | [3] |
| 86 | Oroxylum indicum  (Bignoniaceae) | Shyonaka | Roots  Bark and seeds | Carminative  Diaphoretic  Astringent | [82] |
| 87 | *Passiflora foetida .*L  (Passifloraceae) | Jhumka lata | Whole plant | Antibacterial,  analgesic and antidiarrhoeal | [52] |
| 88 | *Petiveria alliacea.*L  (Phytolaccaceae) | Guinea | Leaves | Anti-inflammatory and antispasmodic | [52] |
| 89 | *Phyllanthus fraternus* G.L.Webster (Phyllanthaceae) | Bhui-amalaki, Tamalki | Whole Plant | Antimicrobial, anti-oxidant, | [53] |
| 90 | *Piper nigrum* L.  Piperaceae | Pippali | Fruits | Digestive, appetizer  and carminative | [3] |
| 91 | *Plumbago zeylanica* L.  Plumbaginaceae | Chitrak | Roots | anti-atherogenic, cardiotonic, hepatoprotective, neuroprotective | [76] |
| 92 | *Prunus armeniaca* L.  Rosaceae | Peetaalu | Kernels | Antitussive,  antiasthmatic | [3] |
| 93 | Psoralea corylifolia  (Fabaceae) | Babchi | Seeds | Laxative  Anti-helminthic | [83] |
| 94 | *Pueraria tuberosa* (Willd.) DC.  Leguminosae | Vidarikand | tubers | permatogenic, immune booster, aphrodisiac, anti-inflammatory, cardiotonic and brain tonic | [76] |
| 95 | *Punica grantum.L*  (Lythraceae) | Dadima | Plant | Anti-inflammatory and antibacterial | [51] |
| 96 | Rhododendron anthopogon  (Ericaceae) | Talisapatra | Stems and leaves | Antitussive  Diaphoretic | [84] |
| 97 | *Rubus occidentalis* L.  Rosaceae | - | Roots | Anti-cancer | [51] |
| 98 | *Rumex hastatus* D. Don  Polygonaceae | Katambal | Roots and bark | Astringent | [3] |
| 99 | *Saussurea lappa* (Decne.) Sch.Bip.  Compositae | Kustha | Roots | anti-inflammatory, analgesic, anti-ageing | [53] |
| 100 | *Scindapsus officinalis*  (Araceae) | Gajpeepal | Fruit | Antioxidant and anti-inflammatory | [52] |
| 101 | *Semecarpus anacardium*  (Anacardiaceae) | Ballataka or bhilwa | Plant | Anti-inflammatory and antioxidant |  |
| 102 | *Solanum nigrum* L.  Solanaceae | Kakamachi | Fruit, whole plant | anti-oxidant, hepatoprotective, anti-ulcerogenic | [53] |
| 103 | *Syzygium aromaticum* (L.) Merr. & L.M.Perry  Myrtaceae | Lavanga | Flower buds | Anti-bacterial, carminative,  anti-cancer,  anti-fungal,  anti-oxidant and anti-inflammatory | [53] |
| 104 | *Tabernaemontana coronaria*  (Apocynaceae) | Chandini | Plant | Antioxidant and  anti-epileptic | [52] |
| 105 | *Taxus bacaata*  (Taxaceae) | Sthauneya | Leaves  Roots  Bark | Antispasmodic,  antifungal and antibacterial | [85] |
| 106 | Tephrosia purpurea  ( Fabaceae ) | Sharapunkha | Roots and whole plant | Anti-diabetic | [86] |
| 107 | *Terminalia chebula* Retz.  Combretaceae | Haritaki | Roots | anti-inflammatory, analgesic | [53] |
| 108 | *Tinospora cordifolia* (Willd.) Miers  Menispermaceae | Guduchi | Stem and leaves | Antipyretic,  antiperiodic,  anti-inflammatory | [3,53,87] |
| 109 | *Trichosanthes dioica* Roxb.  Cucurbitaceae | Patola | Roots and fruits | Cathartic, febrifuge | [3] |
| 110 | *Urtica diocia*  ( Urticaceae) | Vrscikali | Leaves | Antibacterial, antioxidant, analgesic,  anti-inflammatory, antiviral,  anticancer | [51] |
| 111 | Valveriana wallichi  (Caprifoliaceae) | Valeriana jatamansi | Roots  Rhizome  Seed  Flower | Treatment of neurological disorders | [88] |
| 112 | *Vetiveria zizanioides*  ( Poaceae) | Ushira  Or  Khus | Plant | Anti-inflammatory and anti-septic | [89] |
| 113 | *Vitex negundo* L.  (Lamiaceae) | Nirgundi | Leaves, seeds and stem bark | Anti-inflammatory,  analgesic | [3,60] |
| 114 | *Vitex trifolia* L.  (Lamiaceae) | Sinduvaara | Leaves, roots and fruits | Febrifuge,  antibacterial,  anthelmintic,  cytotoxic | [3] |
| 115 | *Withania somnifera*  (L.) Dunal (Solanaceae) | Kakoli, Ashwagandha | Roots | Antimicrobial, aphrodisiac, liver tonic,  anti-inflammatory agent | [90] |
| 116 | *Xanthium strumarium .*L  (Asteraceae) | Gokhra,  Chota datura | Leaves | Antipyretic  And  antibacterial | [52] |
| 117 | *Zanthoxylulm leprieurii*  (Rutaceae) | Tejovati  and  tumbru | Stem bark | Antibacterial,  antifungal,  antitumor and antioxidant | [51] |
| 118 | *Zingiber officinale*  ( Zingiberaceae) | Ardraka | Rhizome, root | Antiarthritis,  anti-inflammatory, antidiabetic, antibacterial, antifungal, anticancer | [51] |

[22] Pilón-Jiménez, B. A., Saldívar-González, F. I., Díaz-Eufracio, B. I., & Medina-Franco, J. L. BIOFACQUIM: A mexican compound database of natural products. Biomolecules, (2019). 9(1).

[23] Bultum LE, Woyessa AM, Lee D. ETM-DB: integrated Ethiopian traditional herbal medicine and phytochemicals database. BMC Complement Altern Med. 2019;19(1):212.

[24] Syahdi RR, Iqbal JT, Munim A, Yanuar A. HerbalDB 2.0: Optimization of construction of three-dimensional chemical compound structures to update Indonesian medicinal plant database.Pharmacogn. J. 2019;11(6):1189-1194.

[25] Afendi FM, Okada T, Yamazaki M, Hirai-Morita A, Nakamura Y, Nakamura K., et al. KNApSAcK Family Databases: Integrated metabolite–plant species databases for multifaceted plant Research. **Plant Cell Physiol**. 2011;(2): e1-e1.

[26] Ashfaq UA, Mumtaz A, Qamar TU, Fatima T. MAPS Database: Medicinal plant activities, phytochemical and structural database. Bioinformation. 2013;9(19):993-995.

[27] Kettner, Kosch, Lang, Lachner, Oborny, Teppan, Creating a Medicinal Plant Database. (2005). 413.

[28] Grafahrend-Belau E, Weise S, Koschutzki D, Scholz U, Junker BH, Schreiber F. MetaCrop: a detailed database of crop plant metabolism. Nucleic Acids Res. 2007;(Database): D954-D958.

[29] Uddin Md. Effective and simple methods of preventing the transmission of viral diseases. J Adv Med Life Sci. 2020; V8I1.02.

[30] Hussain N, Chanda R, Abir RA, Mou MA, Hasan MdK, Ashraf MA. MPDB 2.0: a large scale and integrated medicinal plant database of Bangladesh. BMC Res Notes. 2021;(1).

[31] Hawkins, C., Ginzburg, D., Zhao, K., Dwyer, W., Xue, B., Xu, A., et al. Plant Metabolic Network 15: A resource of genome-wide metabolism databases for 126 plants and algae. J. Integr. Plant. Biol. (2021) 63: 1888– 1905

[32] Kalwij, J.M. Review of ‘The Plant List, a working list of all plant species. J Veg Sci. (2012) 23: 998-1002.

[33] Xue R, Fang Z, Zhang M, Yi Z, Wen C, Shi T. TCMID: traditional Chinese medicine integrative database for herb molecular mechanism analysis. Nucleic Acids Res. 2012;(41): D1089-D1095.

[34] Nguyen-Vo T-H, Le T, Pham D, Nguyen T, Le P, Nguyen A.et al. VIETHERB: A database for vietnamese herbal species. J Chem Inf Model. 2018;(1):1-9

[35] Sekhar, Shailasree. Database of Medicinal Plants from Western Ghats - India. (2015).

[36] Latheef SA, Prasad B, Bavaji M, Subramanyam G. A database on endemic plants at Tirumala hills in India. Bioinformation. 2008;(6):260-262.

[37] Venugopalan Nair SN, Ved DK, Ravikumar K, Tabassum IF, Sureshchandra ST, Somasekhar BS, et al. Indian Medicinal Plants Database (IMPLAD) and threatened medicinal plants of India. In: Conservation and Utilization of Threatened Medicinal Plants: 2020:63-92.

[38] Mohanraj K, Karthikeyan BS, Vivek-Ananth RP, Chand RPB, Aparna SR, Mangalapandi P. IMPPAT: A curated database of Indian medicinal plants, phytochemistry and therapeutics. Sci rep. 2018;(1).

[39] Tota K, Rayabarapu N, Moosa S, Talla V, Bhyravbhatla B, Rao S. InDiaMed: A Comprehensive Database of Indian Medicinal plants for Diabetes. Bioinformation. 2013;9(7):378-380.

[40] Sargia. B, Singh. B, Gupta.N, Gahlot LK, Gulati T, Hasija Y. MED-PDB: An online database of medicinal plants. J Adv Pharm Edu Res 2018;7(4):204-207.

[41] Meetei PA, Singh P, Nongdam P, Prabhu NP, Rathore R, Vindal V. NeMedPlant: a database of therapeutic applications and chemical constituents of medicinal plants from north-east region of India in genomic sequences. Bioinformation. 2012;(4):209-211.

[42] Pathania, S., Ramakrishnan, S. M., Bagler, G. Phytochemica: a platform to explore phytochemicals of medicinal plants. Database. 2015, bav075.

[43] Gradinaru T-C, Petran M, Dragos D, Gilca M. PlantMolecularTasteDB: A database of taste active phytochemicals. Front Pharmacol. Published online 2022.

[44] Kumar A, Kumar R, Sharma M, Kumar U, Gajula M, Singh K. Uttarakhand Medicinal Plants Database (UMPDB): A platform for exploring genomic, chemical, and traditional knowledge. Data. 2018;(1):7.

[45] Usmani SS, Kumar R, Kumar V, Singh S, Raghava GPS. AntiTbPdb: a knowledgebase of anti-tubercular peptides. Database. 2018.

[46] Sharma A, Dutta P, Sharma M, Rajput NK, Dodiya B, Georrge JJ., et al. BioPhytMol: a drug discovery community resource on anti-mycobacterial phytomolecules and plant extracts. J Cheminform. 2014;(1).

[47] Squires B, Macken C, Garcia-Sastre A, Godbole S, Noronha J, Hunt V., et al. BioHealthBase: informatics support in the elucidation of influenza virus host–pathogen interactions and virulence. Nucleic Acids Res. 2007;(suppl_1): D497-D503.

[48] Catanho M, Mascarenhas D, Degrave W, Miranda AB. GenoMycDB: a database for comparative analysis of mycobacterial genes and genomes. Genet Mol Res. 2006;5(1):115-126.

[49] Reddy TBK, Riley R, Wymore F, Montgomery P, DeCaprio D, Engels R., et al. TB database: an integrated platform for tuberculosis research. Nucleic Acids Res. 2009;(Database): D499-D508.

[50] Radusky L, Defelipe LA, Lanzarotti E, Luque J, Barril X, Marti MA., et al. TuberQ: a Mycobacterium tuberculosis protein druggability database. Database. 2014;(0): bau035-bau035.

[51] Sharifi-Rad J, Salehi B, Stojanović-Radić ZZ, Fokou PVT, Sharifi-Rad M, Mahady GB, et al. Medicinal plants used in the treatment of tuberculosis - Ethnobotanical and ethnopharmacological approaches. Biotechnol Adv. Published online 2017:107134.

[52] Verma, D., Mudgal, B., Chaudhary, P., Mahakur, B., Mitra, D., Pant, K., et al. Medicinal plant of Uttarakhand (India) and their benefits in the treatment of tuberculosis: Current perspectives. GJBB, (2020). 9(3), 75-85.

[53] Samal, J., Ayurvedic management of pulmonary tuberculosis: A systematic review. J. Intercult. Ethnopharmacol. 2016 5 (1), 86-91.

[54] Radji, M., Kurniati, M., Kiranasari, A. Comparative antimycobacterial activity of some Indonesian medicinal plants against multi-drug resistant Mycobacterium tuberculosis. J. App. Pharm. Sci. 2015; 5 (1), 019-022.

[55] Alam J, Mujahid Mohd, Badr B, Rahman Md, Akhtar J, Khalid M., et al. An insight of pharmacognostic study and phytopharmacology of Aquilaria agallocha. J Appl Pharm Sci. Published online 2015:173-181.

[56] Prabhu, A., Antimycobacterial activity of certain mangrove plants against multi-drug resistant Mycobacterium tuberculosis. Asian J. Med Sci. 2014 5(3), 54-7.

[57] Tamrakar, V. K., Bhat, J., Parihar, N. S., Rajasubramaniam, S., Thakur, H. In vitro evaluation of anti-mycobacterial active plant extracts against Mycobacterium tuberculosis using mycobacterium growth indicator tubes (MGIT-960) BACTEC system. (2022).

[58] Zhao T, Li C, Wang S, Song X. Green Tea (Camellia sinensis): A Review of Its phytochemistry, pharmacology, and toxicology. Molecules. 2022;(12):3909.

[59] Oladimeji AV, Valan M. Phytochemical profile of cannabis plant: A review. J Pharmacogn Phytochem. 2020;(3):680-687.

[60] Namita, P., Mukesh, R. Medicinal Plants used as antimicrobial agents: review. Int. Res. J. Pharm. 2012; 3, 31-40.

[61] Nkouam GB, Adjoh G, Leudeu CBT, Kouebou C,Tchiegang C, Kapseu C. Physico-chemical properties of fruits, seed and oil of kapok (Ceiba pentandra Gaertn.) tree of different provenances from the northern part of Cameroon. Int. J. Agric. Innov. Res 6 (2017): 275-278.

[62] Alvin, A., Miller, K.I., Neilan, B.A., Exploring the potential of endophytes from medicinal plants as sources of antimycobacterial compounds. Microbiol Res. 2014; 169 (7-8), 483-95.

[63] Mehta A, Srivastva G, Kachhwaha S, Sharma M, Kothari SL. Antimycobacterial activity of Citrullus colocynthis (L.) Schrad. against drug sensitive and drug resistant Mycobacterium tuberculosis and MOTT clinical isolates. J Ethnopharmacol. 2013;(1):195-200.

[64] Dhanabal, S.P., Lall, N., Pavithra, N., Chaitanya, M.V.N.L. Natural products as an important lead for discovery of new antitubercular agents: A review. Int. J. Pharm. Pharm. Sci. 2015; 7(10), 2-7

[65] Rao H, Rao P, Hegde P. A review on Insulin plant (Costus igneus Nak). Pharmacognosy Reviews. 2014;(15):67.

[66] Getachew S, Medhin G, Asres A, Abebe G, Ameni G. Traditional medicinal plants used in the treatment of tuberculosis in Ethiopia: A systematic review. Heliyon. 2022;8(5): e09478.

[67] Changtam C, de Koning HP, Ibrahim H, Sajid MS, Gould MK, Suksamrarn A. Curcuminoid analogs with potent activity against Trypanosoma and Leishmania species. Eur J Med Chem. 2010;45(3):941-956.

[68] Gauli, K., Sharma, S. K., Kunwar, R. M., Bussmann, R. W., Paniagua-Zambrana, N. Y. Cymbopogon citratus (DC.) Stapf. (2021).

[69] Wani IA, Kumar V, Verma S, Tasleem Jan A, Rather IA. Dactylorhiza hatagirea (D. Don) Soo: A Critically Endangered Perennial Orchid from the North-West Himalayas. Plants. 2020; 9(12):1644.

[70] Sharma M, Dhaliwal I, Rana K, Delta AK, Kaushik P. Phytochemistry, Pharmacology, and Toxicology of Datura Species-A Review. Antioxidants (Basel). 2021;10(8):1291.

[71] Singh, S., Parmar, N., & Patel, B. A review on Shalparni (Desmodium gangeticum DC.) and Desmodium species (Desmodium triflorum DC. & Desmodium laxiflorum DC.)–Ethnomedicinal perspectives. J. Med. Plant Res. (2015). 3(4), 38-43.

[72] Kumar S, Das G, Shin HS, Patra JK. Dioscorea spp. (A Wild Edible Tuber): A Study on Its Ethnopharmacological Potential and Traditional Use by the Local People of Similipal Biosphere Reserve, India. Front Pharmacol. 2017; 8:52.

[73] Kumar, R., Meena, R. S., Verma, A. K., Hemant, A., & Panwar, A. Analysis of genetic variability and correlation in fennel (Foeniculum vulgare Mill.) germplasm. Agricultural Research and Technology, 2017; 3(4), 125-129.

[74] Kumaran M.S, Ananthi.A, Vimala T, Sivaselvi P. Molecular docking studies of gloriosa superba for anti-cancer and anti-tuberculosis. World J. Pharm. Res, 2014.2(3), 247–252.

[75] Gupta, B. M., Ahmed, K. M., Gupta, R. Glycyrrhiza glabra (medicinal plant) research: A scientometric assessment of global publications output during 1997-2016. Pharmacognosy Journal, 2018, 10(6).

[76] Gupta, V. K., Kaushik, A., Chauhan, D. S., Ahirwar, R. K., Sharma, S., Bisht, D. Anti-mycobacterial activity of some medicinal plants used traditionally by tribes from Madhya Pradesh, India for treating tuberculosis related symptoms. J. Ethnopharmacol. (2018). 227, 113-120.

[77] Firdous Q, Mohammad FB, Mubashir HM. Ethnopharmacology, phytochemistry and biological activity of Inula racemosa Hook F: A review. Int. J. Ayurveda Res. 2019;9(1):95- 102.

[78] Kadali, V. N., Ramesh, T., Pola, S. R., & Sandeep, B. V. Anti-Tubercular effects of medicinal plants: A short review. pharmacy journal,2016 1(1)1-13.

[79] Poonam K, Singh GS. Ethnobotanical study of medicinal plants used by the Taungya community in Terai Arc Landscape, India. J Ethnopharmacol. 2009;123(1):167-176.

[80] Kaur S, Kumar M, Kumar S. Identification of polyphenols in leaf extracts of Lawsonia inermis L. with antioxidant, antigenotoxic and antiproliferative potential. Int J Green Pharm. 2014;(1):23.

[81] Dalli, M., Bekkouch, O., Azizi, S. E., Azghar, A., Gseyra, N., Kim, B. Nigella sativa L. Phytochemistry and Pharmacological Activities: A Review (2019–2021). Biomolecules, 2021.12(1), 20.

[82] Jagetia GC. "A review on the medicinal and pharmacological properties of traditional ethnomedicinal plant sonapatha, oroxylum indicum." Sinusitis.  2021; 5(1):71-89

[83] Pai FT, Lu CY, Lin CH. Psoralea corylifolia L. Ameliorates Collagen-Induced Arthritis by Reducing Proinflammatory Cytokines and Upregulating Myeloid-Derived Suppressor Cells. Life (Basel). 2021;11(6):587

[84] Innocenti, G., Dall’Acqua, S., Scialino, G., Banfi, E., Sosa, S., Gurung, K., et al. Chemical composition and biological properties of Rhododendron anthopogon essential oil. Molecules, 2010. 15(4), 2326-2338.

[85] Indhumathi, M., Shanuvas A., “Anti-Tubercular Activity of Indian Medicinal Plants – A Review.” American Journal of Pharmacy and Health Research (2018) 6.3: 1–20.

[86] Mangwani N, Singh PK, Kumar V. Medicinal plants: adjunct treatment to tuberculosis chemotherapy to prevent hepatic damage. J Ayur Int Med. 2019; S0975-9476(18):30705-30708.

[87] Dhanabal, S.P., Lall, N., Pavithra, N., Chaitanya, M.V.N.L. Natural products as an important lead for discovery of new antitubercular agents: A review. Int. J. Pharm. Pharm. Sci. 2015, 7(10), 2-7.

[88] Li, J., Li, X., Wang, C., Zhang, M., Ye, M., Wang, Q. The potential of Valeriana as a traditional Chinese medicine: traditional clinical applications, bioactivities, and phytochemistry. Frontiers in Pharmacology. (2022).

[89] Snigdha. M, Kumar, Mohapatra S.S, Sharmistha, Deepa, C. An overview on Vetiveria zizanioides. Res. J. Pharm. Biol. Chem. Sci. (2013). 4. 777-783.

[90] Adaikkappan, P., Kannapiran, M. and Anthonisamy, A. Anti-Mycobacterial Activity of Withania somnifera and Pueraria tuberosa against Mycobacterium tuberculosis H37Rv. J. Acad. ind. res. (2012) 1, 153- 15.
